# Case report: A rare case of sintilimab-induced gastric stenosis and literature review

**DOI:** 10.3389/fonc.2023.1091459

**Published:** 2023-01-25

**Authors:** Kunkun Song, Haoxu Dong, Shujun Jiang, Xiaohu Xu, Chao Zhang, Qian Chen, Qi Wang

**Affiliations:** ^1^ Department of Integrated Traditional Chinese and Western Medicine, Tongji Hospital, Tongji Medical College, Huazhong University of Science and Technology, Wuhan, China; ^2^ Department of Pathology, Tongji Hospital, Tongji Medical College, Huazhong University of Science and Technology, Wuhan, China; ^3^ Department of Gastroenterology, Tongji Hospital, Tongji Medical College, Huazhong University of Science and Technology, Wuhan, China

**Keywords:** programmed death-1, sintilimab, immune-related adverse events, gastric stenosis, non-small cell lung cancer

## Abstract

Sintilimab is a fully human IgG4 monoclonal antibody against programmed death-1 (PD-1) used to treat classical Hodgkin’s lymphoma and various solid tumors. With increasing use of sintilimab, some rare adverse reactions have been reported. Here, we report a case of a 50-year-old woman with squamous non-small cell lung cancer (NSCLC) (metastasis to pericardium and pleura) who received two cycles of 200 mg sintilimab immunotherapy combined with albumin-bound paclitaxel and carboplatin chemotherapy and one cycle of sintilimab monotherapy. She was diagnosed with Sjogren’s syndrome (with symptoms of fever, dry mouth, dysphagia, and eating difficulty) after three cycles’ treatment and received standard steroidal therapy. Prior to admission, the patient experienced severe stomach discomfort with vomiting and was hospitalized. Upper gastrointestinal iodine angiography showed significant gastric stenosis as well as lower esophageal stenosis. Subsequent ultrafine gastroscopy revealed ulceration at the stenotic site and an absence of normal peristalsis of the gastric wall. Pathological examination of the lesions showed reactive changes, including ulceration, fibrosis, and inflammatory cell infiltration. After multidisciplinary consultation, it was considered that the patient’s gastric stenosis with inflammatory fibrosis changes was due to a sintilimab-induced immune hyperinflammatory reaction. The patient had been treated with standard steroidal therapy since suffering from Sjogren’s syndrome, but the gastric stenotic changes were not relieved. The patient then received regular bouginage of esophago-cardiac stenosis under gastroscopy to physically reexpand the fibrous hyperplasia and stenotic site, enabling normal eating function. To our knowledge, this is the first case of gastric stenosis in a patient with squamous NSCLC after using sintilimab and may help clinicians better understand potential immune-related adverse events due to sintilimab and improve assessment and management.

## 1 Introduction

Over the last decade, significant progress has been made in the use of anti-programmed death-1 (PD-1)/programmed death-ligand 1 (PD-L1) immunotherapy for treating cancer. Cancer cells can take advantage of PD-1 signaling to escape immune surveillance (1). By specifically blocking PD-1 on the surface of T cells, anti PD-1/PD-L1 immunotherapy can eliminate tumor-mediated immune suppression, thereby restoring the ability of T cells to recognize and destroy tumor cells. Anti PD-1 monotherapy, or its combination with other chemotherapy, has shown promising anti-tumor efficacy in clinical applications with an increased survival rate and, overall, manageable and acceptable adverse side effects. As anti PD-1/PD-L1 immunotherapy shows good tolerability and patient compliance, its clinical use is increasing. Sintilimab is a fully human IgG4 monoclonal antibody against PD-1 that is approved for treating classical Hodgkin’s lymphoma and various solid tumors, including hepatocellular carcinoma, non-small cell lung cancer (NSCLC), esophageal squamous carcinoma, and gastric adenocarcinoma (2). Clinical trials for other malignancies are on-going. With increasing use, some rare adverse reactions have emerged. Thus, it is necessary that clinicians are aware of all possible side effects for early identification and successful treatment. Similar to other anti PD-1 therapies, the main side effects of sintilimab are immune-related adverse events (irAEs), which occur because blocking PD-1 on T cells can decrease the body’s immune tolerance and cause autoimmune activation in some people. Reported irAEs of sintilimab include immune associated pneumonia, colonitis, hepatitis, nephritis, and myocarditis; however, sintilimab-induced gastric lesions are uncommon. Here, we describe a rare case of gastric stenosis in a patient with squamous NSCLC after using sintilimab.

## 2 Case presentation

A 50-year-old woman with a history of squamous NSCLC and metastasis to pericardium and pleura, who had pathological tissue samples that were ALK negative and PD-L1 positive with Tumor cell Proportion Score (TPS) of 80%, received two cycles of 200 mg sintilimab immunotherapy combined with chemotherapy of albumin-bound paclitaxel and carboplatin (from July to August 2020) and one cycle of sintilimab monotherapy (September 2020). The patient developed symptoms of fever, thirst, dysphagia, and difficulty eating, as well as severely damaged submandibular gland (no response to acid stimulation) and lacrimal gland (a tear flow rate of zero in both eyes) function after three cycles’ treatment. She was diagnosed with Sjogren’s syndrome through consultation with an immunologist in September 2020 and discontinued sintilimab immunotherapy. The patient was then treated with steroidal therapy (80 mg methylprednisolone). The symptoms of fever and thirst were relieved and food intake started to increase one week later. The steroid dose was gradually reduced (60 mg methylprednisolone for 10 days, 40 mg for 14 days, 20 mg for 11 weeks, and 16 mg for 2 weeks prior to admission). While treating Sjogren’s syndrome, four cycles of albumin-bound paclitaxel mono-chemotherapy were administered (from October 2020 to January 2021).

**Figure 1:**
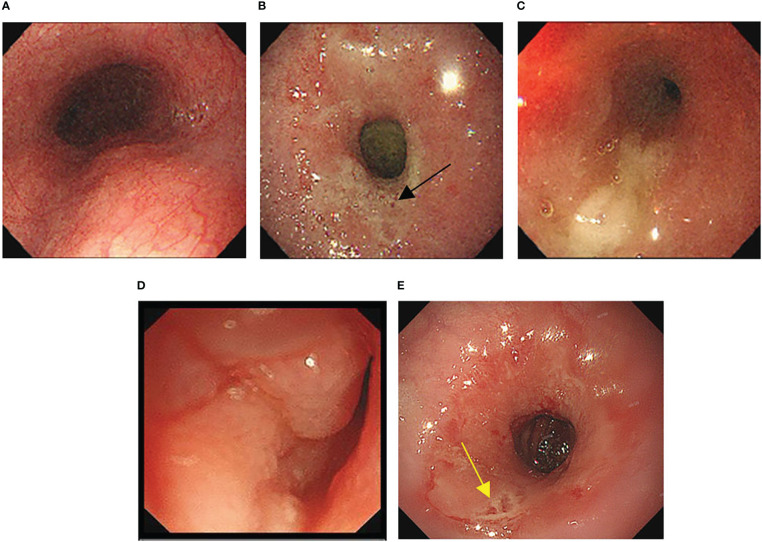
(A-C) Morphology of esophagus under gastroscopy. **(A)** The upper esophagus was morphologically normal. **(B)** Circumferential stenosis was observed 34cm from the esophageal incisor (black arrow indicates lower esophageal ulcers). **(C)** Extreme stenosis was observed 37cm from the esophageal incisor so that the endoscope could not pass through. **(D, E)** Gastric morphology under ultrafine endoscopy. **(D)** The gastric body was covered with white moss, and easy to bleed while touching. **(E)** Normal peristalsis was absent and ulcers were seen in the gastric body (yellow arrow indicates gastric body ulcers).

**Figure 2:**
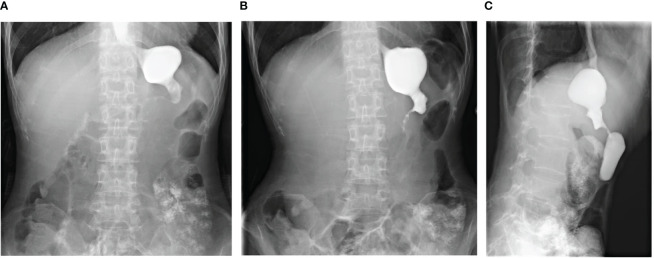
Upper gastrointestinal iodine angiography showed significant stenosis of the gastric cavity. **(A, B)** Anteroposterior views of the upper gastrointestinal iodine angiography. **(C)** Lateral views of the upper gastrointestinal iodine angiography.

**Figure 3:**
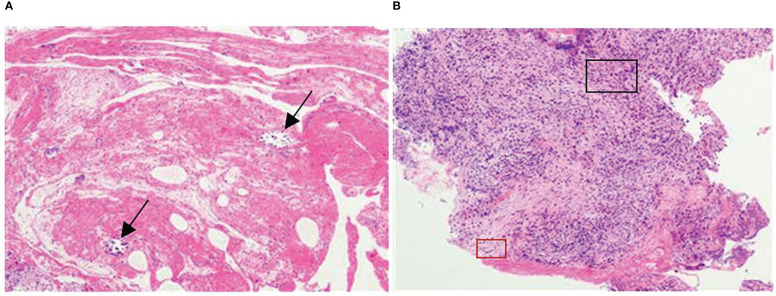
Pathological manifestation at the site of stenosis. **(A)** A patch of smooth muscle tissue with small foci of calcifications (arrows indicate the calcifications). **(B)** A patch of small blood vessels, nerve and hyperplasia of fibrous tissue with inflammatory cell infiltration (inflammatory and fibrous cells were scattered, black andred rectangles indicate relatively obvious inflammatory and fibrous cell aggregates, respectively).

For approximately 10 days prior to admission in February 2021, the patient experienced heavy stomach discomfort and irregularly vomited her stomach contents, although there was no significant abdominal pain, with regular intestinal exhaust and defecation, without symptoms of fever, diarrhea, dizziness, headache, or backache. On physical examination, there was no obvious abdominal tenderness or rebound pain. Additional relevant examinations were performed after admission. Blood tests showed red blood cells of 3.57×10^12^/L, hemoglobin of 10^6^ g/L, D-D dimer of 1.1 μg/mL FEU, creatinine of 35 μmol/L (white blood cells count, platelet count, glutamic pyruvic transaminase, glutamic oxalacetic transaminase and urea nitrogen were normal). Fecal occult blood was weakly positive. Gastroscopy was performed under anesthesia by a gastroenterologist. Circumferential stenosis of the lower esophagus was observed approximately 34–37 cm from the esophageal incisors (**Figures 1A–C**), with a brittle mucous membrane and tendency to bleed while touching; the remaining esophageal mucosa was smooth with a clear vascular texture. As the endoscope could not pass through the stricture at that time, the gastric lesion was not clear. Upper gastrointestinal iodine angiography was recommended by the gastroenterologist, which showed an irregular shape of the stomach and obvious gastric stricture (**Figure 2**) with gastroesophageal reflux. To observe the gastric lesion, ultrafine gastroscopy was performed. The gastric cavity was obviously constricted with a peripheral ulcer and normal peristalsis was absent. The surface of the gastric wall was covered with white moss and bled easily while touching, with ulcers visible in the gastric body (**Figures 1D, E**). To further identify the etiology and nature of gastric and esophageal stenosis, ulcer tissue samples were taken for pathological examination. The results showed a patch of smooth muscle tissue with small foci of calcifications, and a patch of small blood vessels, nerve, and hyperplasia of fibrous tissue with inflammatory cell infiltration, which were considered to be reactive changes (**Figure 3**); no carcinoma was found. Positron emission tomography–computed tomography (PET/CT) examination was also performed and showed that the gastric wall the of corpus gastricum was thickened at the site of stenosis, but without obvious metabolism. Thus, cancer was not considered.

Considering the patient’s history of Sjogren’s syndrome after using sintilimab along with the gastroscopy and gastrointestinal iodine angiography results (obvious constriction and stiffness of the gastric cavity, absence of normal peristalsis) and pathological manifestation of inflammatory hyperplasia with fibrosis, after multidisciplinary consultation, the patient’s gastric stenosis with inflammatory fibrosis changes was determined to be caused by a sintilimab-induced immune hyperinflammatory reaction.

**Figure 4:**
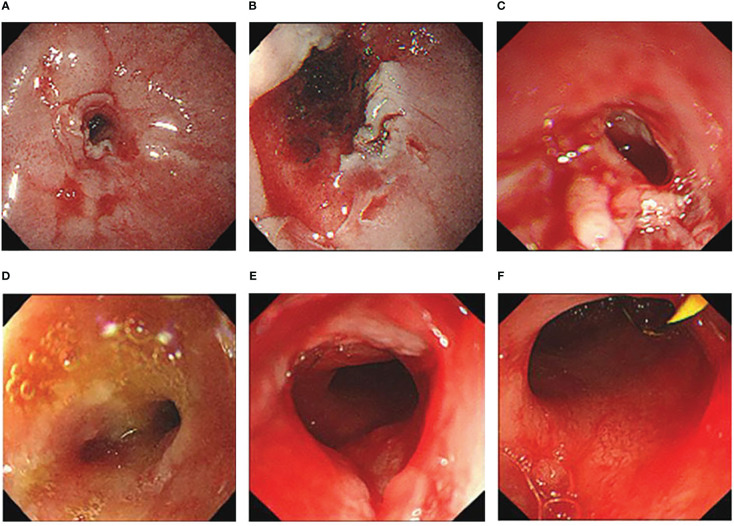
**(A–C)** Cardiac morphology before **(A)**, during **(B)** and after **(C)** laser incision. **(D–F)** Cardiac morphology before **(D)**, during **(E)** and after **(F)** bouginage.

The patient had been treated with steroidal therapy since suffering from Sjogren’s syndrome. As the gastric stenotic changes were not relieved, this complication was not sensitive to steroidal therapy. To relieve vomiting and increase the patient’s energy level, gastroscopic nasointestinal tube placement was performed. Five months later (July 2021), when the patient’s nutritional status improved, laser incision of the stenosis site under gastroscopy was performed (**Figures 4A–C**). Seven days later, a metal rack was placed to expand the stenosis site. The patient could eat normally after these procedures. In February 2022, the metal rack fell off in the stomach and was removed under gastroscopy. Bouginage of the esophago-cardiac stenosis was performed (**Figures 4D–F**); 5 mm, 7 mm, 9 mm, and 11 mm zebra guidewires were used for continuous expansion for 1 min each, with an interval of 1 min. After the operation, the mucosa was obviously torn without active bleeding. An attempt to insert another metal rack failed because the front end of the rack could not pass through the stenosis. Although the metal rack was not implanted, the patient was able to eat normally after bouginage. Subsequently, the patient underwent regular bouginage of esophago-cardiac stenosis approximately every 2–3 months (second time in May 2022, third time in July 2022, and fourth time in October 2022). The patient was able to eat normally during this period.

## 3 Discussion

At present, lung cancer accounts for 11.4% of new cancers, second only to breast cancer (11.7%), and results in 18% of cancer-associated fatalities globally, making it the leading cause among all cancer types (3). More than 85% of lung cancers are the NSCLC type, of which approximately 33% are the squamous subtype (4). For patients with unresectable NSCLC who do not have targeted gene mutations, platinum-based doublet chemotherapy was once the first-line treatment option. However, in the last decade, the development of immune checkpoint inhibitors (ICIs) has greatly changed this situation. Among all ICIs, anti PD-1/PD-L1 immunotherapy is the most mature method. Sintilimab is a Chinese domestic monoclonal antibody against PD-1 that was initially approved by the National Medical Products Administration (NMPA) to treat patients with classical Hodgkin’s lymphoma who had already received more than two lines of systematic chemotherapies but the disease was still progressive (5, 6). With ongoing clinical trials, the use of sintilimab has gradually expanded to other solid tumors. For NSCLC, sintilimab immunotherapy combined with chemotherapy has been approved by NMPA as the first-line treatment (7). Specifically, sintilimab combined with gemcitabine and platinum is approved for squamous NSCLC, while sintilimab combined with pemetrexed and platinum is approved for EGFR-negative and ALK-negative non-squamous NSCLC.

Although immunotherapy has achieved great success, there are increasing reports of treatment-related adverse events (TRAE) induced by ICIs. Thus, this issue cannot be ignored (8). A meta-analysis of 36 phase II/III trials showed a pooled incidence of TRAEs of 54%–76% (9). Another study assessed the lethal toxicity spectrum associated with ICIs, finding that the incidence of lethal TRAEs was 0.361% for PD-1 inhibitors and 0.63% for PD-L1 inhibitors (10). For sintilimab, the pooled incidences of TRAEs and lethal TRAEs are 16.7%–100% and 1%–6.25%, respectively (2). TRAEs caused by ICIs are mainly irAEs. By enhancing the ability of T cells to destroy tumors cells, ICIs can promote cancer patients’ immune function; however, if activation of the immune system is aggressive, irAEs may occur (11). Common sintilimab-induced irAEs include debilitation, fever, pneumonia, hypothyroidism, skin eruptions, and thrombocytopenia. Less common sintilimab-induced irAEs include hypoadrenocorticism, cardiotoxicity and myocarditis, paraneoplastic syndrome, and rhabdomyolysis (2). However, immune-induced gastric lesions are uncommon. Thus, this case of sintilimab-induced irAEs of gastric stenosis with inflammatory fibrosis changes is rare.

Follow-up of PD-1 monotherapy in patients with neoadjuvant resectable NSCLC showed that patients with high PD-L1 expression (TPS ≥ 50%) tended to have a higher two-year disease-free survival rate (12). PD-L1 immunochemical staining and targeted DNA sequencing of tumor samples from 29 NSCLC patients before immunotherapy revealed a positive correlation between TPS and the degree of pathological regression. In addition, a higher TPS (≥ 50%) was significantly associated with major pathological response (13). The patient in the present case had high PD-L1 expression (TPS = 80%), suggesting that anti PD-1 immunotherapy has a high response rate and sintilimab is in accordance with the indications for its application.

The patient developed Sjogren’s syndrome after three cycles of sintilimab treatment, and obvious gastric stenosis was found five months later. Due to the long time since sintilimab was discontinued, gastroscope pathological examination and PET/CT examination was performed; tumor-associated lesions of the stenosis area were not considered.

In addition to sintilimab, the patient had also received chemotherapy of albumin-bound paclitaxel and carboplatin. However, the main adverse reactions of albumin-bound paclitaxel are leukopenia, neutropenia, neuropathy, fatigue, and infection (14), and that of carboplatin are myelosuppression, anaphylaxis, hepatotoxicity, ototoxicity, and cardiotoxicity (15). The pathological manifestation of inflammatory hyperplasia with fibrosis of this patient were more consistent with the characteristic of hyperimmunity, which was the main side effect of ICIs. Considering the fact that the patient had a history of irAEs (Sjogren’s syndrome), it was thought that the patient’s gastric stenosis with inflammatory fibrosis changes was caused by a sintilimab-induced immune hyperinflammatory reaction.

This patient had suffered from dry mouth and swallowing and eating discomfort since Sjogren’s syndrome was diagnosed and these symptoms were relieved by steroidal therapy. Thus, the symptom of eating discomfort was always considered to be related to Sjogren’s syndrome. Gastroscope examination was not performed until 5 months later because of increasingly discomfort after eating accompanied by vomiting, which finally revealed obvious gastric stenosis with ulcer and fibrosis. Our experience suggests that if patients experience stomach discomfort or difficulty eating after using sintilimab, gastroscopy should be performed as soon as possible to detect any gastric lesions and allow adjustment of the dose or discontinuation.

Glucocorticoids remain the first-line treatment for irAEs. Patients with severe reactions or reactions involving important organs might also require biological immunomodulators. Individualized treatment is recommended for irAEs of different organs caused by ICIs (16). Although our patient was treated with standardized steroidal therapy due to Sjogren’s syndrome for 5 months (80 mg methylprednisolone for 7 days, 60 mg for 10 days, 40 mg for 14 days, 20 mg for 11 weeks, and 16 mg for 2 weeks prior to admission), the lesion of gastric stenosis was not reversed. This result suggests that immune-related gastric stenosis might not respond well to steroidal therapy, or that the dose of glucocorticoid used in this case was insufficient or not applied early enough. Regular bouginage of esophago-cardiac stenosis was performed under gastroscopy to physically reexpand the fibrous hyperplasia and stenotic site, which enabled normal eating function for the patient. This case suggests one approach for the treatment of gastric stenosis secondary to immunotherapy, although more research is still needed.

## 4 Conclusion

This is the first reported case of gastric stenosis caused by sintilimab. Since sintilimab monotherapy or combination therapy is widely used for various malignant tumors, it is essential to understand the potential irAEs and conduct adequate evaluation and management.

## Data availability statement

The original contributions presented in the study are included in the article/supplementary material. Further inquiries can be directed to the corresponding author.

## Ethics statement

The studies involving human participants were reviewed and approved by Tongji Hospital, Tongji Medical College, Huazhong University of Science and Technology. The patients/participants provided their written informed consent to participate in this study. Written informed consent was obtained from the individual(s) for the publication of any potentially identifiable images or data included in this article.

## Author contributions

KS: Designed the case report and wrote the manuscript. HD: Collected the patient’s data. SJ: Edited the image. XX: Collated and verified the data. QW: Revised and reviewed the manuscript. CZ: Analyzed the pathological results. QC: Edited and Analyze the digestive endoscopy images. All authors contributed to the article and approved the submitted version.

## References

[1] Yi M Zheng X Niu M Zhu S Ge H Wu K . Combination strategies with PD-1/PD-L1 blockade: Current advances and future directions. Mol cancer (2022) 21(1):28. doi: 10.1186/s12943-021-01489-2 35062949 PMC8780712

[2] Liu X Yi Y . Recent updates on sintilimab in solid tumor immunotherapy. biomark Res (2020) 8(1):69. doi: 10.1186/s40364-020-00250-z 33292551 PMC7708241

[3] Sung H Ferlay J Siegel RL Laversanne M Soerjomataram I Jemal A . Global cancer statistics 2020: GLOBOCAN estimates of incidence and mortality worldwide for 36 cancers in 185 countries. CA: Cancer J Clin (2021) 71(3):209–49. doi: 10.3322/caac.21660 33538338

[4] Shi Y Chen W Li C Zhang Y Bo M Qi S . Efficacy and safety of first-line treatments with immune checkpoint inhibitors plus chemotherapy for non-squamous non-small cell lung cancer: A meta-analysis and indirect comparison. Ann palliative Med (2021) 10(3):2766–75. doi: 10.21037/apm-20-1498 33549014

[5] Hoy SM . Sintilimab: First global approval. Drugs. (2019) 79(3):341–6. doi: 10.1007/s40265-019-1066-z 30742278

[6] Zhang L Mai W Jiang W Geng Q . Sintilimab: A promising anti-tumor PD-1 antibody. Front Oncol (2020) 10:594558. doi: 10.3389/fonc.2020.594558 33324564 PMC7726413

[7] Zhang L Lin W Tan F Li N Xue Q Gao S . Sintilimab for the treatment of non-small cell lung cancer. Biomark Res (2022) 10(1):23. doi: 10.1186/s40364-022-00363-7 35436956 PMC9014583

[8] Ramos-Casals M Brahmer JR Callahan MK Flores-Chávez A Keegan N Khamashta MA . Immune-related adverse events of checkpoint inhibitors. Nat Rev Dis primers (2020) 6(1):38. doi: 10.1038/s41572-020-0160-6 32382051 PMC9728094

[9] Xu C Chen YP Du XJ Liu JQ Huang CL Chen L . Comparative safety of immune checkpoint inhibitors in cancer: Systematic review and network meta-analysis. BMJ (2018) 363:k4226. doi: 10.1136/bmj.k4226 30409774 PMC6222274

[10] Wang DY Salem JE Cohen JV Chandra S Menzer C Ye F . Fatal toxic effects associated with immune checkpoint inhibitors: A systematic review and meta-analysis. JAMA Oncol (2018) 4(12):1721–8. doi: 10.1001/jamaoncol.2018.3923 PMC6440712 30242316

[11] Naidoo J Page DB Li BT Connell LC Schindler K Lacouture ME . Toxicities of the anti-PD-1 and anti-PD-L1 immune checkpoint antibodies. Ann oncol: Off J Eur Soc Med Oncol (2015) 26(12):2375–91. doi: 10.1093/annonc/mdv383 PMC6267867 26371282

[12] Gao S Li N Gao S Xue Q Wang S Lv F . Two-year follow-up of single PD-1 blockade in neoadjuvant resectable NSCLC. J Clin Oncol. (2021) 39(15_suppl):8522. doi: 10.1200/JCO.2021.39.15_suppl.8522

[13] Wang S Yuan P Mao B Li N Ying J Tao X . Genomic features and tumor immune microenvironment alteration in NSCLC treated with neoadjuvant PD-1 blockade. NPJ Precis Oncol (2022) 6(1):2. doi: 10.1038/s41698-021-00244-6 35027673 PMC8758728

[14] Nakao M Fujita K Suzuki Y Arakawa S Sakai Y Sato H . Nab-paclitaxel monotherapy for relapsed small cell lung cancer: Retrospective analysis and review. Anticancer Res (2020) 40(3):1579–85. doi: 10.21873/anticanres.14105 32132060

[15] Oun R Moussa YE Wheate NJ . The side effects of platinum-based chemotherapy drugs: a review for chemists. Dalton Trans (2018) 47(19):6645–53. doi: 10.1039/C8DT00838H 29632935

[16] Esfahani K Elkrief A Calabrese C Lapointe R Hudson M Routy B . Moving towards personalized treatments of immune-related adverse events. Nat Rev Clin Oncol (2020) 17(8):504–15. doi: 10.1038/s41571-020-0352-8 32246128

